# Experimental Study of the Guided Wave Directivity Patterns of Thin Removable Magnetostrictive Patches

**DOI:** 10.3390/s20247189

**Published:** 2020-12-15

**Authors:** Akram Zitoun, Steven Dixon, Graham Edwards, David Hutchins

**Affiliations:** 1 Brunel Composites Centre, College of Engineering, Design and Physical Sciences, Brunel University London, London UB8 3PH, UK; 2 Monitoring and Inspection Research, TWI Ltd., Granta Park, Cambridge CB21 6AL, UK; Graham.Edwards@twi.co.uk; 3 Department of Physics, The University of Warwick, Coventry CV4 7AL, UK; s.m.dixon@warwick.ac.uk; 4 School of Engineering, The University of Warwick, Coventry CV4 7AL, UK; d.a.hutchins@warwick.ac.uk

**Keywords:** magnetostriction, guided wave modes, Lamb waves, shear horizontal waves

## Abstract

The characteristics of removable magnetostrictive thin patches are investigated for the generation of guided waves in plates. The directivity patterns of SH, S0 and A0 modes have been measured in a thin metallic plate for different combinations of static and dynamic magnetic field directions. This used different coil geometries such as racetrack and spiral coils to generate the dynamic magnetic field, as well as separate biasing static magnetic fields from permanent magnets. This arrangement generated signals via both Lorentz and magnetostrictive forces, and the resultant emitted guided waves were studied for different dynamic and static magnetic field directions and magnitudes. It is demonstrated that different guided wave modes can be produced by controlling these parameters.

## 1. Introduction

Non-Destructive Testing (NDT) techniques are widely used for the inspection of different structures in numerous industrial applications. Guided Waves (GWs) are among these techniques and they are widely used in different applications such as the inspection of pipes [1,2], rails [3,4] and plates [5,6]. GWs in plates and pipes are complex as they are generally dispersive, although some modes, such as the SH0 mode in a plate and the T(0,1) mode in a pipe, can be non-dispersive. Their characteristics are affected by the material properties and the geometry of the structure within which the elastic wave is propagating. GW inspection is a long-range NDT technique, potentially allowing large areas to be inspected in situations where there is limited access [7,8], and has applications in the aerospace sector, for wing inspection, for example. Guided waves can be used to detect different types of defects in plate-like structures, including delaminations [9,10] in composites. The possibility of damage detection in aerospace composite has been investigated using both S0 and SH0 modes [11]. The work in this paper concentrates on these two modes, although A0 modes can also be useful because of their smaller wavelength.

GWs can be generated using various transduction mechanisms such as piezoelectric transducers and Electro-Magnetic Acoustic Transducers (EMATs). The latter are attracting more industrial interest as they are contact-free transducers and do not need any couplant to operate on metal surfaces. The main transduction mechanism for EMATs is the Lorentz force, although magnetostriction can sometimes play a part, for example on corroded steel [12,13]. By reconfiguring the orientation of coils and applied static magnetic fields, they are able to generate and detect different types of guided wave modes [14]. One of the major limitations of conventional EMATs is that the Lorentz force can only operate on conductive samples, and their efficiency is heavily affected by the material properties and lift-off [15]. EMATs typically have a low efficiency when compared to piezoelectric transducers [16,17]. Air-coupled ultrasound is another non-contact technique that can be used for either through-transmission or GW operation [18], although control of alignment can be an issue for GW work. Note that pulse compression processing or other signal processing methods are often required to improve signal to noise ratios [19].

To deal with the efficiency of EMATs, and to overcome some of the limitations of air-coupled systems, this paper investigates the use of removable magnetostrictive patches that are attached to the surface of the specimen. While magnetostrictive patch transducers (MPTs) have been reported in the literature [17,20], this paper extends the knowledge in this area by systematically studying the variables that affect their operation, and also studies the properties of the resulting GW generation. The removable concept was to allow these MPTs to operate at surfaces on which a more permanent adhesive could not be used. An example is for aircraft wing inspection, where operators would not allow a strong adhesive, which might damage the surface in any way, to be used.

Magnetostriction can be defined as the change in the dimensions of a sample when an external magnetic field is applied. It was first discovered by Joule [21], and occurs when an external magnetic field is applied to a ferromagnetic material [22,23,24]. The magnetostrictive coefficient is usually written as *λ*, and is defined as the ratio of the ratio of the fractional change in length δ*l* over the initial length *l_0_*

(1) $$\lambda =\frac{\delta l}{l_{0}}.$$

**Figure 1:**
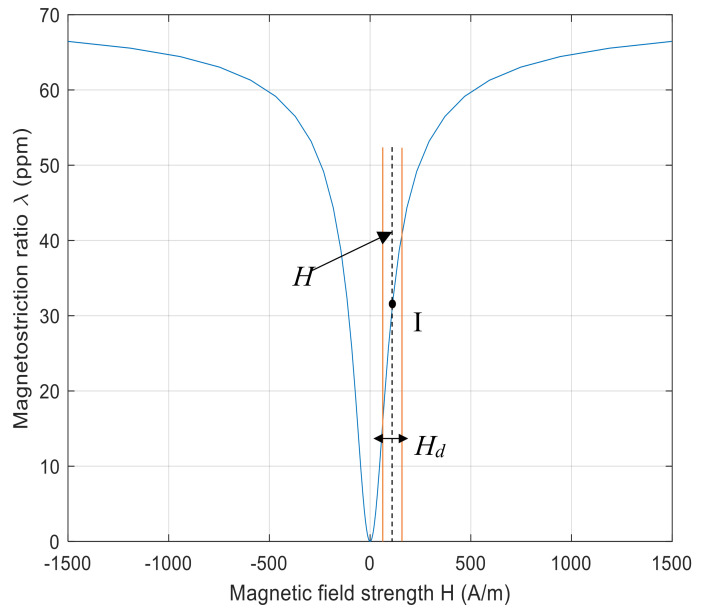
Magnetostriction curve (**blue line**) for an iron-cobalt alloy with the operating range of the applied dynamic field indicated by the vertical brown lines.

In the absence of an applied magnetic field, the different magnetic domains are orientated in such a way as to minimise the total magnetization of the sample [25]. If a sufficiently strong external magnetic field is applied to the sample in one of these directions, the magnetic dipole moments tend to align with the applied field, but in doing so they induce strain into the lattice. The degree of strain induced depends on the specific sample properties, including material composition and microstructure [26]. When an external magnetic field is applied, the change in dimensions that occurs is known as the Joule effect; conversely, when an external strain is applied, the change in magnetization is known as the Villari effect [24,27,28,29,30,31]. There is a non-linear relationship between the applied field (*H*) and *λ*, as illustrated in Figure 1 for an iron–cobalt alloy. In practice, it is preferable to ensure that the link between the external applied magnetic field and the generated strain through magnetostriction is optimised by operating around the steepest part of the strain-applied field curve, and it is also helpful that, in this region of the curve, the response is approximately linear. The static magnetic biasing field *H_s_* is selected so the generated strain is created at some bias point (I), such as that shown in Figure 1. The applied dynamic field (*H_d_*) then causes the resultant change in dimensions.

When a dynamic magnetic field is applied, the *λ*–*H* curve causes linear oscillation around the bias point, providing the highest strain change for a given field change. The process of selecting the biasing magnetic field and the oscillating dynamic field is of importance, as a linear response is required when magnetostrictive patch transducers are used to generate guided waves [29,32]. This is because a non-linear response can induce harmonic frequencies of the main drive frequency, making it more difficult to selectively generate a particular guided wave mode. The idea behind choosing the bias point in the position shown is that the largest variation in strain is obtained for the minimum applied dynamic magnetic field, as this is the region of both greatest slope as well as of optimum linearity. Magnetostriction has been used in this way by various authors for ultrasonic generation in a range of materials—see, for example, [33,34,35,36,37,38].

Magnetostrictive Patch Transducers (MPTs) [32] can be used in different shapes and forms for different applications. For example, omnidirectional MPTs have been developed, which use a cylindrical permanent magnet placed on top of a circular coil [20]. Directional patches have also been developed with a U-shape permanent magnet placed on top of a meander coil [39]. These designs were able to generate different wave modes, but a detailed understanding of the generation mechanisms was not reported. In this paper, various combinations (directions, amplitude, etc.) of static and dynamic magnetic fields have been studied, so as to determine the mechanisms that influence operation of the magnetostrictive patch, and hence which arrangement is optimum for a particular guided wave mode. One example is in the likely magnitude and direction of the forces in an MPT that might result from combinations of different coil geometries and static magnetic field directions. Note that both Lorentz and magnetostrictive forces will be generated in our experiments. The Lorentz force acts on electrons, whereas the physical origin of the magnetostriction is the magnetization–strain coupling caused by the quantum spin–orbit interaction. The magnitude and direction of each will influence the directional characteristics and relative generation efficiency of SH0, S0 and A0 modes. This is studied in this paper, which gives an understanding of how different static and dynamic magnetic field directions with respect to the removable patch on the surface of a flat plate can affect the generation of different guided wave modes.

While MPTs have been reported previously in the literature, earlier papers did not present detailed characteristics such as guided wave directivity patterns for different configurations of both dynamic and magnetic fields. The work described in this paper provides experimental measurements of the characteristics of guided waves when generated by a removable magnetostrictive patch. This is studied for various conditions of coil and magnet geometries, including one where the contribution from the Lorentz mechanism is minimised by careful choice of the orientation of dynamic and static magnetic fields.

## 2. Experimental Setup

Two sets of experiments were performed. The first was to study vibrational patterns directly underneath the removable patches, while the second measured the directivity patterns of SH, S0 and A0 modes at some distance from the patch.

### 2.1. Vibrational Modes Directly under the Patch

These initial experiments were designed to investigate the vibrational characteristics of the region within a 3 mm thick glass plate that was directly below a thin magnetostrictive patch when subject to different dynamic and static magnetic fields. The patch was adhered to the plate surface using a thin layer of double-sided tape and could be removed after a particular measurement without damaging the surface of the sample. These first experiments were conducted using a glass plate, as this was known to be isotropic and non-conducting and have low acoustic attenuation properties over the frequency range of interest. The lack of electrical conductivity was felt to be important, as the presence of conductivity might have affected the Lorentz forces that would also be generated within the MPTs in some configurations.

**Figure 2:**
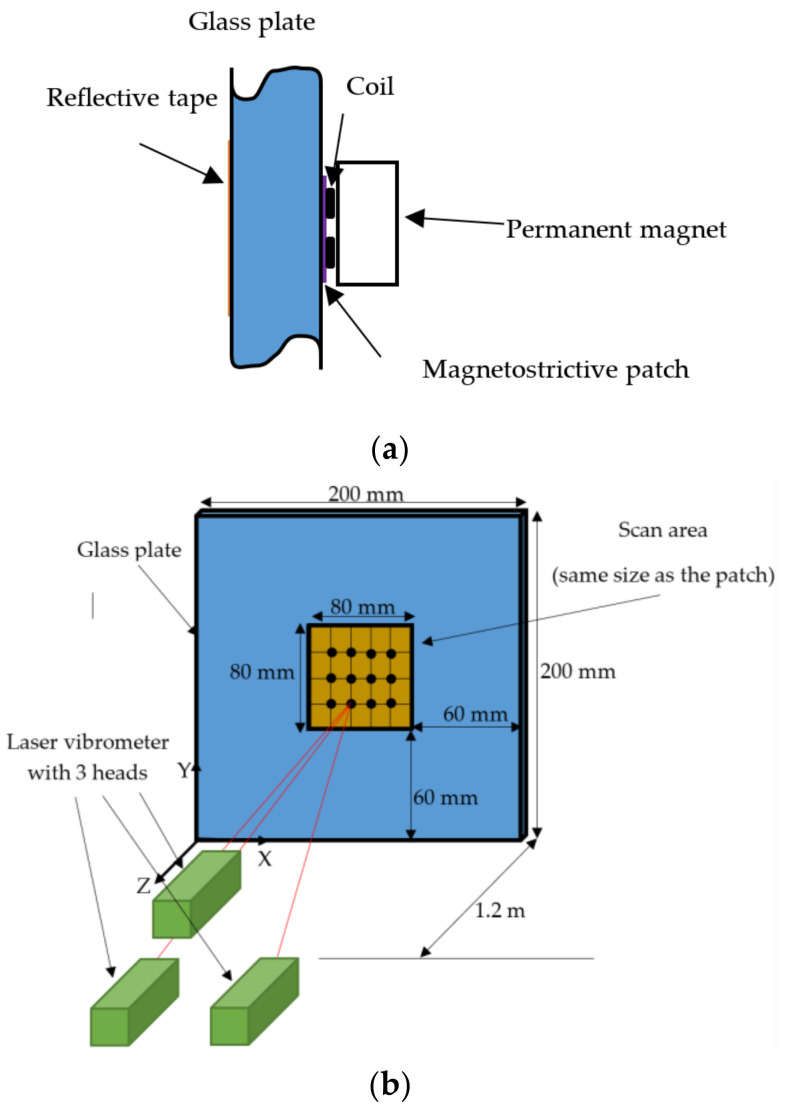
(**a**) A cross-section of the measurement setup, showing the relative location of the coil, permanent magnet and magnetostrictive patch making up the MPT, plus the reflective tape on the far surface of the glass plate; (**b**) Schematic diagram of the three vibrometer heads (green) incident upon the reflective tape (orange) which was attached to the far side of the glass plate (blue).

A schematic diagram of the experimental configuration used for this type of measurement is presented in Figure 2. The magnetostrictive patch was a VACOFLUX 48 iron cobalt alloy (^®^VACUUMSCHMELZE GmbH & Co., Hanau, Germany) with a saturation magnetization of 2.35 T and high permeability value of up to 18,000 N A^−2^. The mechanical and magnetic properties of the VACOFLUX iron-cobalt alloy are given in Table 1.

An 80 × 80 mm square patch of 55 µm thickness was attached at the centre of the glass plate of dimensions 200 × 200 mm. On the other side of the glass plate, and directly under the patch, a reflective tape was attached in order to reflect light back to a vibrometer, used to detect the vibrations of ultrasonic waves that existed directly under the MPT. A PSV-400 3D vibrometer laser scanner within the vibrometer setup was placed parallel to the glass plate at a distance of 1.2 m. The laser head is constructed with three laser beams within a single vibrometer head. They detect vibrations both in-plane and out-of-plane. Each beam is responsible for measuring the vibration in a different direction (X, Y and Z). The distance was selected in order to obtain an optimal reading from the laser beam while keeping a good scanning resolution. The scanning heads of the PSV-400 system were positioned so as to directly observe the vibrations of the patch after excitation.

**Figure 3:**
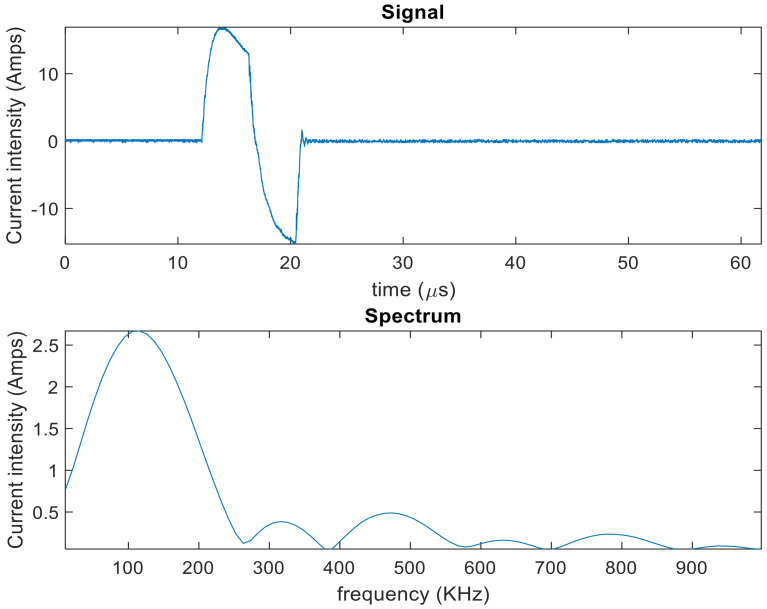
The current waveform from the high-power pulsing unit that was injected into the excitation coil above the removable MPT, showing both the current waveform (**top**) and the corresponding frequency spectrum (**bottom**).

In order to stimulate the magnetostrictive patch to generate GWs, either a racetrack coil or a pancake coil were connected to a high-power pulsing unit. The current waveform that was input to the racetrack coil consisted of a one-cycle sine wave of 120 kHz central frequency (Figure 3). The operation frequency of 120 kHz was chosen initially due to the need to ensure that the frequency response was well within the higher frequency limit available from the magnetostrictive material. In order to capture the key results, the patches were scanned and two specific time steps. The first dataset was captured when the excitation signal from the pulsing unit and passing through the coil reached its peak—this was to capture the vibration on the patch while minimising any side vibration or electrical pick-up. Data were also collected at a later time, close to the end of the excitation waveform.

**Figure 4:**
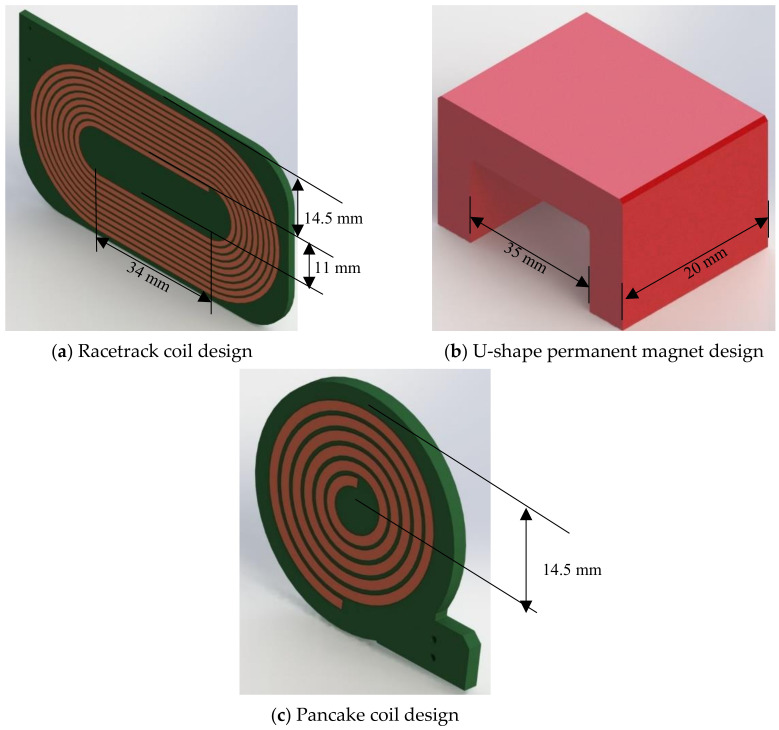
Graphical illustration of (**a**) the racetrack coil, (**b**) the horseshoe permanent magnet and (**c**) the pancake coil.

The coil generating the dynamic magnetic field within the magnetostrictive patch transducer was constructed in one of two configurations, each with a permanent magnet to generate the static magnetic field (*B_s_*) and a coil to provide the dynamic magnetic field (*B_d_*). The racetrack coil shown in Figure 4a, *B_s_* was generated using a U-shape alnico permanent magnet (Figure 4b) in order to ensure that the field was directed in-plane with regards to the patch, i.e., parallel to the plate surface. This permanent magnet was placed so that the field was located at the linear region of a racetrack coil, used to generate the dynamic magnetic field. The racetrack coil consisted of 10 turns, of a 1 mm wide wire with a 0.5 mm spacing between each turn (Figure 4a). A circular pancake coil geometry was also used (Figure 4c), in conjunction with both the horseshoe magnet described above, but also with a cylindrical permanent magnet that generated a static field perpendicular to the plane of the coil. The alnico grade 5 U-shape permanent magnet (Figure 4b) had a remanent magnetic field equal to 1.1 T. The generated permanent magnetic field was measured to be 210 mT in air at the mid-point between the poles of the U-shape magnet, but of course the magnetic flux density would be higher in the patch when it was located between the poles. The cylindrical magnet provided an out-of-plane biasing magnetic field (i.e., perpendicular to the sample surface), and had a remanent magnetic field equal to 1.3 T. The measured generated field at the flat surface of the cylindrical permanent magnet in air was equal to 300 mT.

The main aim of these experiments was to determine the patch vibrational modes for different coil geometries and magnetic field directions. For this reason, a set of four main configurations were studied, and these are presented in Table 2. 

**Figure 5:**
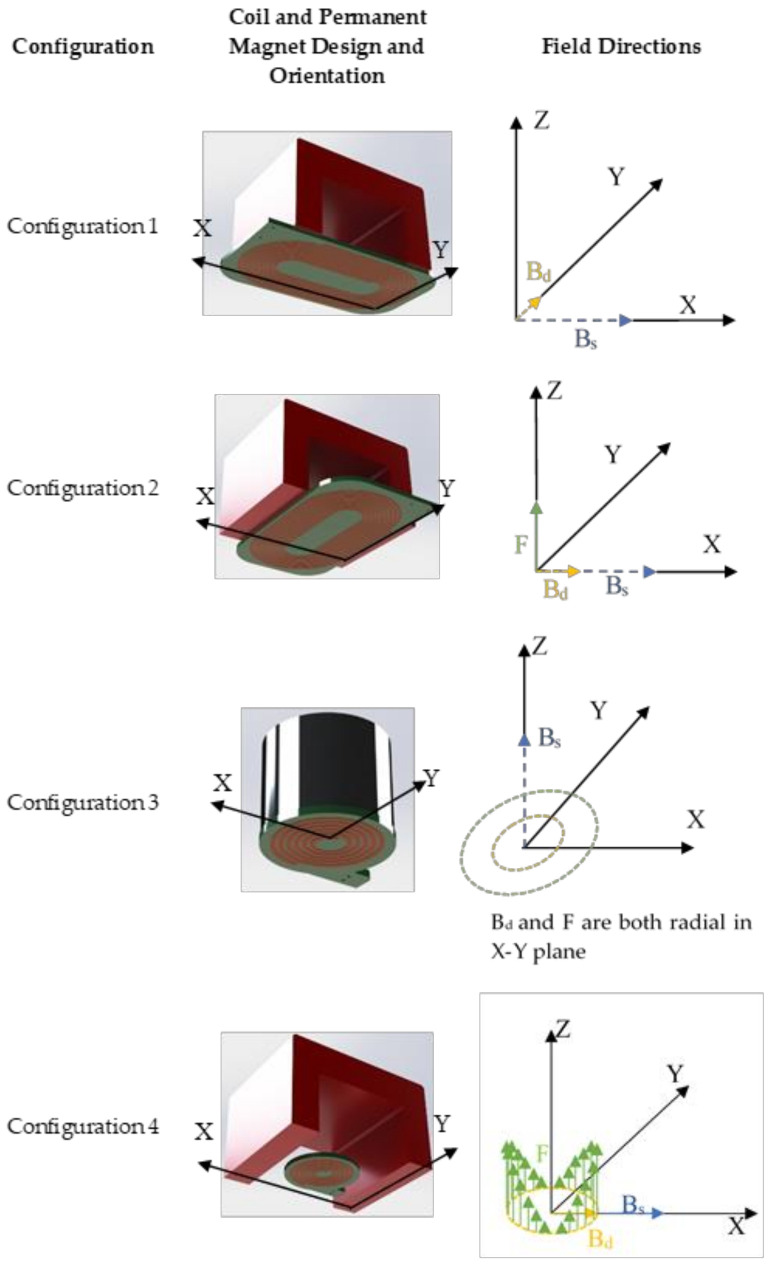
Illustration of the different EMATs for different configuration and the directions of bias field, dynamic field and Lorentz forces if generated.

The four configurations are illustrated more fully in Figure 5. As shown earlier in Figure 2, the X–Y plane contained the plate sample and the patch, with the *Z*-axis being perpendicular to the plate. The configurations were designed to allow a study of the relative interactions between the directions of both *B_s_* (generated by the magnet) and *B_d_* (generated by the coil) for two coil geometries. Note that this was likely to be very different from a standard EMAT using Lorentz forces only, as a substantial magnetostrictive component would be expected, whose magnitude and direction of operation had to be determined. For these reasons, Configurations 1 and 2 have two measurements where the coils/magnet orientation was fixed, but where the whole arrangement could be rotated by 90° with respect to the patch. This would identify any directional effects or anisotropy caused by the patch itself. 

Configuration 3 had an out-of-plane *B_s_* but with the pancake coil in-plane; this configuration would thus be expected to generate both Lorentz and magnetostrictive forces, which would act radially. Conversely, Configuration 4 used an in-plane magnetic field, which would be expected to create a more complicated set of forces normal to the patch via Lorentz forces, as shown.

### 2.2. Guided Wave Mode Generation

**Figure 6:**
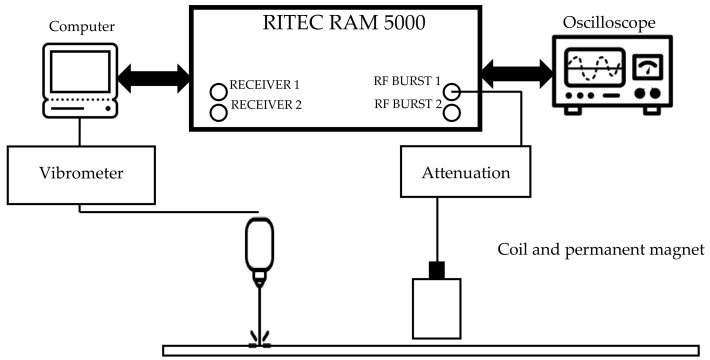
Experimental apparatus used for studying guided wave generation.

**Figure 7:**
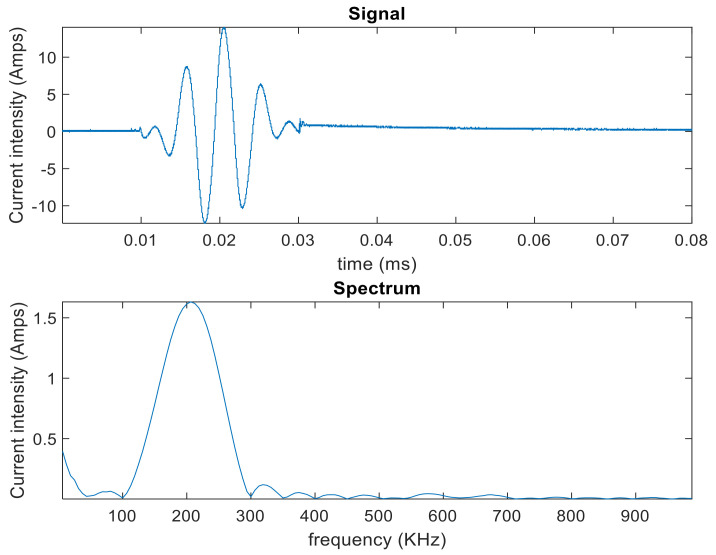
Current waveform from the RITEC RAM-5000 for GW experiments.

The second set of experiments investigated the signals that would be generated by the magnetostrictive patch attached to a metallic sample. The experimental arrangement is shown in Figure 6. In this case, the input was a windowed three-cycle excitation at a 200 kHz central frequency generated by using a RITEC RAM5000 unit. The frequency and the number of cycles were different from the previous experiment set, in order to enhance the signal reading and the signal-to-noise ratio. This combination of frequency/number of cycles allowed a clearer received signal containing S0, SH0 and A0 modes, and allowed a more accurate measurement of the time of flight and, hence, phase velocity of each mode. A higher frequency of 200 kHz was also used for guided-wave generation in the metallic plate, as this would more closely resemble the frequencies that might be used in an NDE experiment. The data-capturing window was 400 µs long. An initial delay of 10 µs to the pulsed signal to be generated through the RITEC system was imposed. The coil impedance was measured using an impedance analyser in order to calculate the root mean squared amplitude of the alternating current driving the coil, which was 15 A. The signal and the magnitude Fast-Fourier transform (FFT) are presented in Figure 7.

The magnetostrictive patch was attached to a 3 mm thick, non-magnetic metallic plate for these guided-wave propagation experiments. The size of the square iron cobalt alloy patch was 76 × 76 mm^2^, and the patch thickness was 55 µm. In these experiments, the coil was a racetrack printed circuit coil of 0.035 mm thickness. The printed coil was insulated from the magnetostrictive patch using a 0.1 mm thick adhesive plastic film. The width of the single track of racetrack coil was 1 mm and the spacing between tracks was equal to 0.5 mm. The coil was formed of 10 turns and it was printed on a single layer of the printed circuit board (PCB). The full coil track width was 14.5 mm, and the PCB substrate was 1.57 mm thick. The permanent magnet was placed on the straight region of the racetrack coil, as previously shown in Figure 5. The design and the dimensions were chosen so that the width of the racetrack coil at its linear portion would be smaller than that of the patch in order to avoid any interaction between the electromagnetic fields and the patch edges.

**Figure 8:**
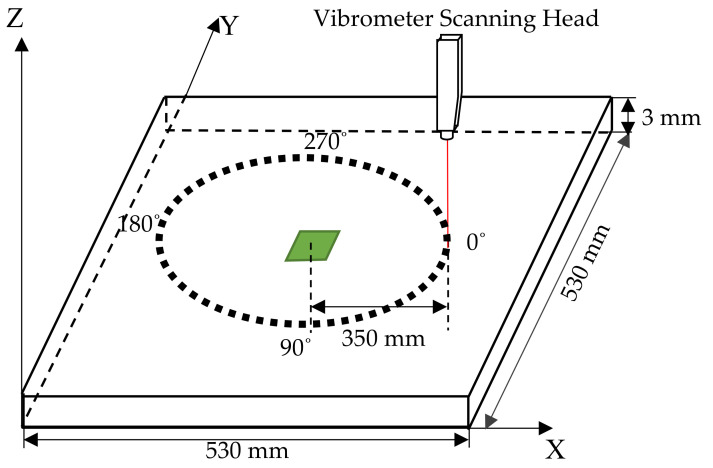
Geometry of scan to measure GW directivity patterns.

For this set of experiments, the detection system consisted of a 3D laser vibrometer mounted on a three-axis arm. The laser head contained three laser beams. The beams are separate 3 Doppler vibrometers providing measurement in the three-axis system for the back scattered light. The optical sensors with the different beams can detect and measure the vibration in the in-plane and the out-of-plane directions. The motion of the laser beam was designed to perform a 360° rotational motion with a 10° angle step so as to capture the waveforms at different angles. The laser beam head was positioned as shown in Figure 8 at a distance of 350 mm from the centre of the patch. The laser system measured the three components of the travelling guided waves in the X, Y and Z directions, with the laser beam normal to the metal plate surface. The fixed distance of the laser vibrometer at various angle to the patch was controlled by the three-axis arm.

Different configurations for the static and dynamic magnetic fields acting on the patch were tested. As before, Table 1 summarizes the different configurations and their orientations with regards the laser vibrometer. This was performed for both racetrack and pancake coil shapes. In Configurations 1, 2 and 4, two measurements ((a) and (b)) were taken with the same coil/magnetic field arrangement but rotated by 90° with respect to the patch. This was to determine the presence of any anisotropy/directional effects within the patch itself. It should be noted that multiple measurements of the same directivity pattern showed good repeatability, with a relatively low error for each measurement point.

## 3. Results

The results of experiments involving both scanning the area under the patch on a glass plate and measuring directivity patterns of GWs at some distance away in a metallic plate are presented in this section. The aim was to show both how the patch vibrated, and how the guided wave modes were generated by the different dynamic and static field configurations.

### 3.1. Vibration Detection Directly Underneath the Patches

#### 3.1.1. Configuration 1

**Figure 9:**
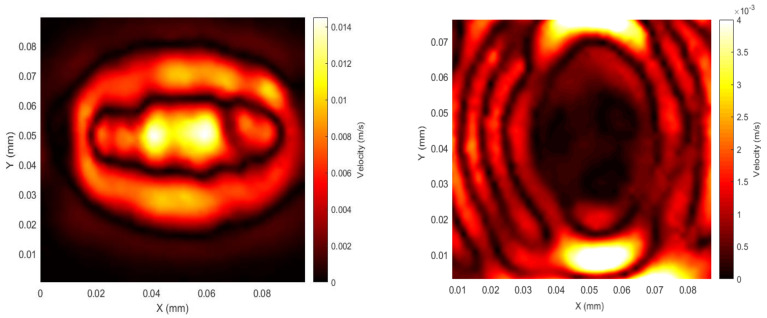
Patch spatial scan for the magnitude of the surface velocity at the beginning of the first peak of the signal (left, *t* = 14 µs) and the end of the pulsed wave (right, *t* = 22 µs) for configuration 1; *f* = 120 kHz, 1 cycle sine wave.

Configuration 1 had the in-plane static (*B_s_*) and dynamic (*B_d_*) fields along the X and Y directions, respectively, with the linear part of the racetrack coil aligned with the X direction. No Lorentz force was expected, as *B_s_* was parallel to the coil direction. The observations for both configurations thus show the contribution of the magnetostriction forces to the generation of the elastic waves. The distribution of vibrations within the patch at both the beginning and end of the signal are presented in Figure 9. As can be seen, the maximum vibration occurred just below the racetrack coil at a time corresponding to 14 µs after the drive current started, and with a similar shape to that of the coil. The scans below represent the magnitude of the surface velocity captured by the laser beam as defined by Equation (2). For these and subsequent measurements, the vibrometer was used to detect motion in all three orthogonal directions (X, Y and Z) with amplitudes of *A_x_*, *A_y_* and *A_z_*, respectively. The overall amplitude of surface vibration at any particular location could then be calculated as the Modulus using the equation

(2) $$Modulus\left(A\right)\;=\;\sqrt{\left(A_{X}{}^{2}+\;A_{Y}{}^{2}+\;A_{Z}{}^{2}\right)}$$

The data captured at the end of each signal show that the waveform has expanded outwards within the patch. The wave has an elliptically shaped wavefront. Other experiments demonstrated that little difference occurred in amplitude or directivity when the magnet/coil assembly was rotated by 90° with respect to the patch. This would be the expected result if there was no preferred magnetostriction direction within the patch. In all cases, the rolling direction, and hence the expected direction of anistopy within the magnetostrictive patch, was parallel to the *Y* axis. This result was seen with other configurations, confirming that anisotropy within the patch did not appear to have a significant effect on the wave modes generated. For all the scans performed directly under the patch, the waves we are looking at are not strictly guided waves at this point and neither are they simple, through thickness bulk waves travelling perpendicular to the sample surface. We can, however, make some crude assumptions about the likely efficiency of these wave arrivals for generating particular wave modes, as we are measuring their amplitude and direction on the opposite side of the sample to where the generation source is located.

#### 3.1.2. Configuration 2

**Figure 10:**
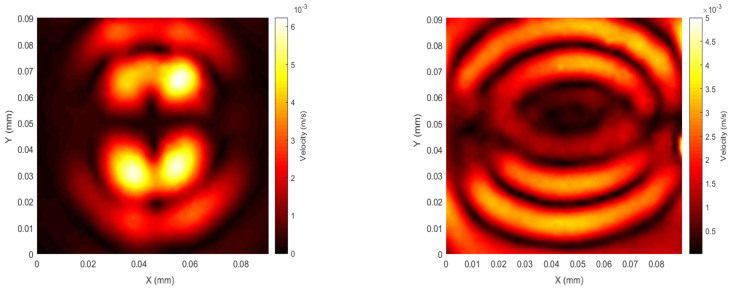
Top: Configuration 2—patch spatial scan at the beginning of the first peak of the signal (**left**, *t* = 14 µs) and the end of the pulsed wave (**right**, *t* = 22 µs); *f* = 120 kHz, 1 cycle sine wave.

The next configuration was designed to set both the dynamic and static fields in the same X direction, as illustrated in Figure 6. The results are presented in Figure 10. The scan captured after a short time delay shows a maximum amplitude for a wavefront heading predominantly in the Y direction, perpendicular to that of *B_d_* and *B_s_*. Lorentz forces, would be expected to be generated in the Z direction, perpendicular to the patch, whereas magneotstrictive forces are generated within the the (X, Y) plane. Thus, for SH0 and S0 GW modes, the Lorentz force would be expected to be of less importance in tems of elastic wave generation. However, this arrangement would be expected to favour SH0 mode amplitudes, as the forces would be all in the X direction, and propagation appears to be predominantly in the Y direction.

#### 3.1.3. Configuration 3

**Figure 11:**
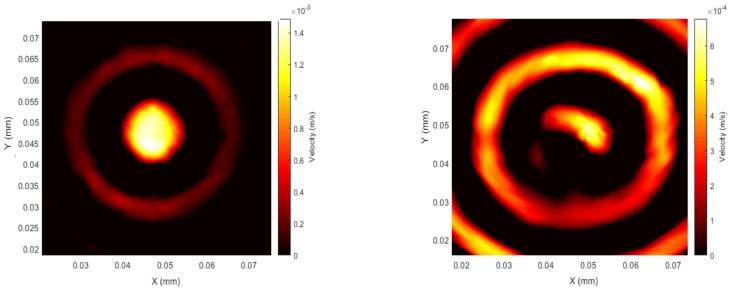
Configuration 3—patch spatial scan at the beginning of the first peak of the signal (**left**, *t* = 14 µs) and the end of the pulsed wave (**right**, *t* = 22 µs); *f* = 120 kHz, 1 cycle sine wave.

Configuration 3 consisted of a permanent magnet, which provided an out-of-plane static magnetic field and a pancake-shaped coil in order to generate an in-plane dynamic field in all radial directions (see Figure 5). For this configuration, both radial Lorentz and magnetostrictive forces would be expected, and these would be in phase. As can be seen from Figure 11, the wave is travelling fairly uniformly in all directions within the patch, and at the beginning of the signal, the first vibration is generated just below the circular coil.

#### 3.1.4. Configuration 4

**Figure 12:**
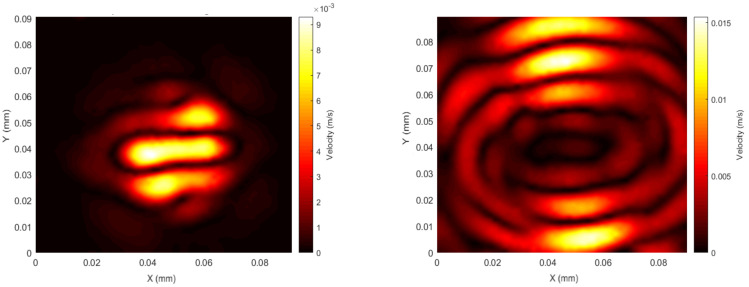
Configuration 4—patch spatial scan at the beginning of the first peak of the signal (**left**, *t* = 14 µs) and the end of the pulsed wave (**right**, *t* = 22 µs); *f* = 120 kHz, 1 cycle sine wave.

Configuration 4 consisted of the U-shape Alnico magnet that was used to generate a static magnetic field along the X direction and a circular coil generating a dynamic radial in-plane field. The results for Configuration 4 are shown in Figure 12, where again it can be seen that the initial vibration distribution is asymmetric, even though a circular pancake coil was used, with more energy radiating in the Y direction (perpendicular to *B_d_* and *B_s_*).

Configuration 4 creates a more complex Lorentz force behaviour. The configurations consist of a linear static magnetic field combined with a circular distribution of dynamic magnetic field and hence a circular distribution of the current vector. This non-uniformity results in Lorentz forces generated along the Z axis with a non-uniform magnitude. The energy generated at early time delays is concentrated in a central location where the Lorentz forces are expected to be minimal (along the Y axis). The GWs are radiated primarily in the Y direction.

### 3.2. Directivity Measurements in a Stainless Steel Plate

**Figure 13:**
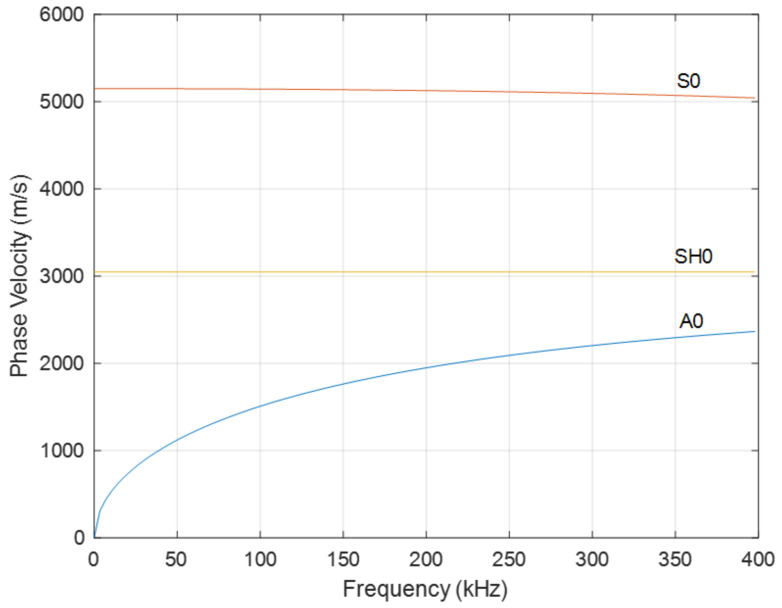
Predicted GW dispersion curves for a 3 mm thick stainless-steel plate.

The next set of experiments were designed to capture GW waveforms as a function of propagation direction once they had propagated a certain distance from the patch. The sample chosen was a 3 mm thick, non-magnetic metallic stainless-steel plate. The aim was to look at the directivities of S0-, SH0- and A0-guided wave modes, these being the three modes allowed in the thin plate for the frequencies of interest. The aim was to see how they were affected by the relative directions of *B_d_* and *B_s_* for a given coil geometry. For such a plate, the expected dispersion curves are shown in Figure 13, and the velocity of three modes at 200 kHz are shown in Table 3.

**Figure 14:**
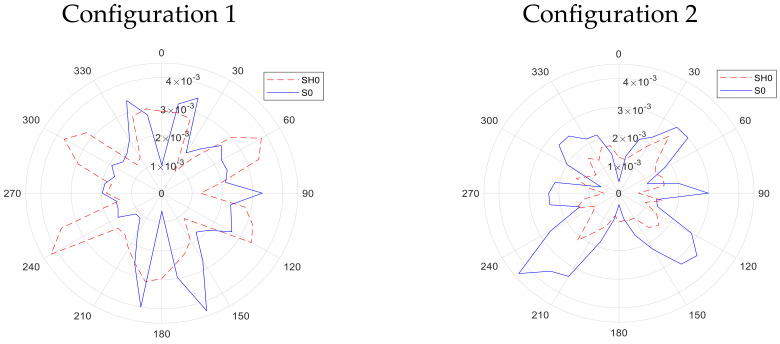

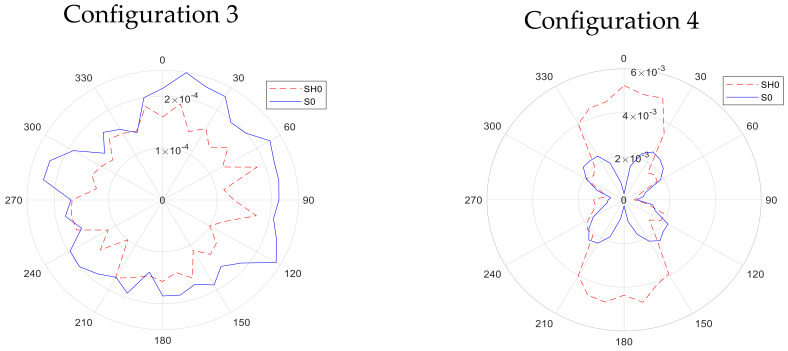
Directivity patterns for S0 and SH0 modes generated on the metallic plate for Configurations 1, 2, 3 and 4 as labelled in the figure.

**Figure 15:**
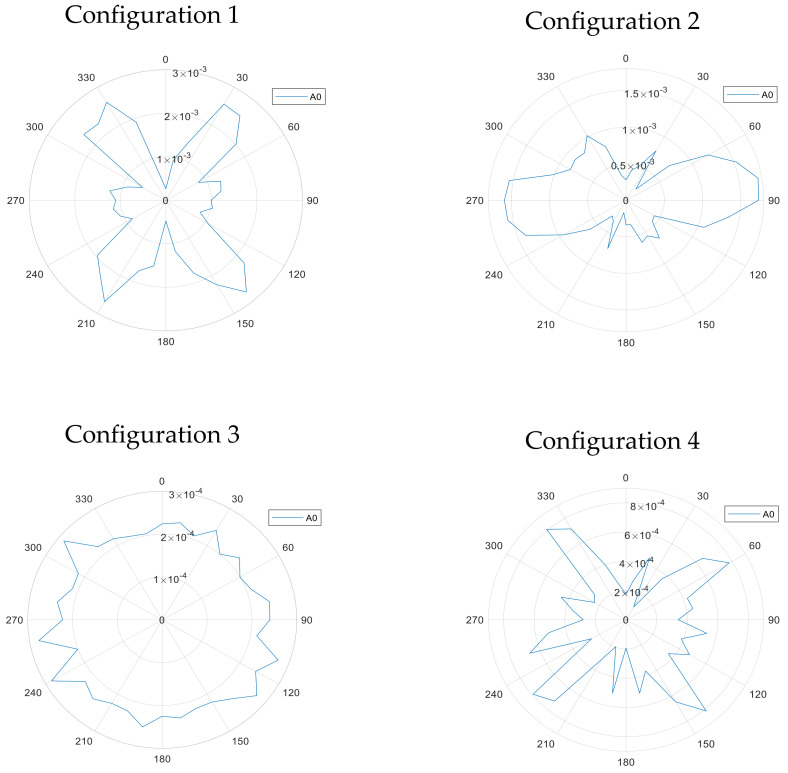
Directivity patterns for the A0 mode generated on the metallic plate for the four different configurations (1, 2, 3 and 4 as labelled in the figure).

The directivity results obtained for the S0 and SH0 modes are shown in Figure 14 for configurations 1–4, using the arrangement shown earlier in Figure 9. The corresponding patterns for the A0 mode are presented in Figure 15. In all cases, the rolling direction of the magnetostrictive patch was at 0° on these diagrams, parallel to two sides of the square patch. It can be seen that the form of the directivity patterns are different for each configuration.

First consider Figure 14 for S0 and SH0 modes. For configuration 1, the directivity pattern for both modes is complicated, with no clear single directivity lobe in a single direction. Their relative amplitudes are similar. It is likely that this behaviour arises from the complex situation observed earlier in Figure 9 from measurements under the patch. Note that this is due predominantly to magnetostriction mechanisms only, as Lorentz forces are expected to be minimal. In configuration 2, the S0 mode is of a higher amplitude than the SH0 mode, but both present a complicated directivity pattern of a similar shape, with multiple lobes. For configuration 3, both the S0 and SH0 modes are fairly uniform in directivity, with the SH0 mode having a slightly higher amplitude. This form of directivity would be expected from the fact that the resultant forces would be expected to be radial in the X–Y plane (see Figure 5). In configuration 4, it can be seen that the SH0 mode is the dominant feature as a bipolar pattern along the *Y*-axis (at 0° and 180°). This is expected, as Bs is acting along the *X*-axis, favouring SH0 generation, noting that initial radiation along this axis was observed earlier to be initiated in the image of Figure 10 (the patch is initially stretched along the *X*-axis, favouring the generation of shear motion leading to the SH0 mode). The S0 has a quadrapolar directivity of smaller amplitude overall, but with lobes rotated with respect to the SH0 mode.

Figure 15 shows the directivity patterns of the A0 mode, again for the four configurations. For configuration 1, the spatial distribution shows the existence of prominent lobes along the 35°–215° and 320°–140° axes. An even more complicated pattern was observed for configuration 2, with multiple lobes. It should be noted that Lorentz forces are being generated in the out-of-plane direction and hence should favour the A0 mode, but despite this, the amplitude is an order of magnitude lower than that of the S0 and SH0 modes, suggesting that magnetostriction is a more efficient generation mechanism. A low amplitude was again observed for configuration 3, where the A0 mode is generated with a fairly uniform directivity, noting that both Lorentz and magnetostriction forces are generated in-plane. For configuration 4, the A0 wave modes radiation plot contains two main lobes at 90° and 270°. The pancake coil and the U-shaped magnet create a non-uniform distribution of the Lorentz force, which is maximum at 90°, explaining the A0 high amplitude at the same angle. At angles such as 0° and 180°, the Lorentz forces are minimal and thus the A0 mode is generated at lower amplitudes.

It was observed in the directivity measurements of Figure 14 and Figure 15 that rotation of the square patch by 90° had no significant effect on the observed directivity patterns. This is evidence for the fact that anisotropy with the patch itself was not likely to contributing significantly to the observed behaviour.

**Figure 16:**
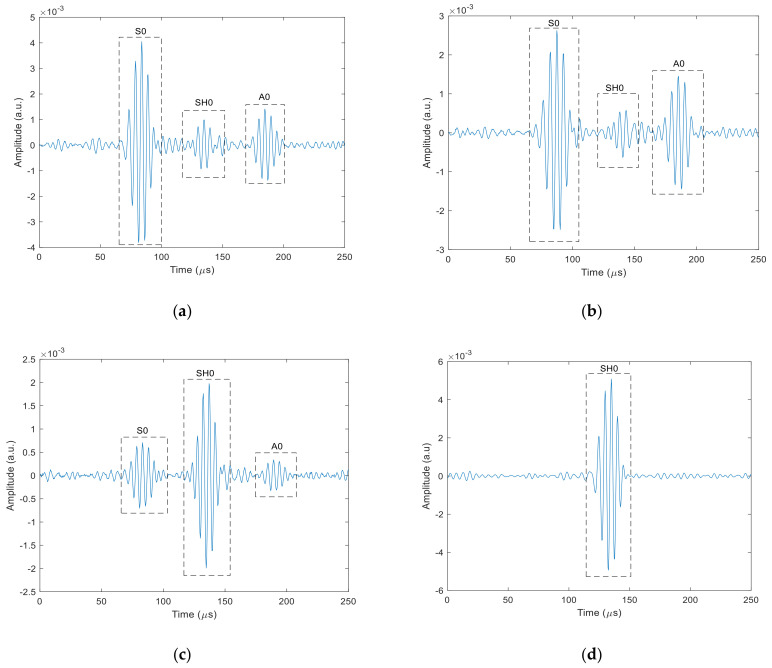
(**a**–**d**): Examples of waveforms detected by the laser vibrometer at a distance of 0.35 m in the stainless steel plate for Configurations 1–4, respectively, at 1 160°, 2 at 90°, 3 at 90° and 4 at 0° respectively.

To illustrate the types of waveforms detected, examples for Configurations 1–4 are presented in Figure 16a–d, respectively, for detection at a distance of 350 mm from the patch centre. The input signal consisted of a three-cycle tone burst signal with a central frequency of 200 kHz. It can be seen that signals corresponding to the three modes (S0, SH0 and A0) were identified based on the velocities expected from the dispersion curves of Figure 13, which are listed in Table 3. In three of the cases, Figure 16a–c, all three modes (S0, SH0 and A0) were detected at the specific radiation angles indicated. For Configuration 4 at an angle of 0°, only the SH0 mode is visible, as might be expected due to the prominent lobe in the SH0 directivity, and the lack of energy radiated by the A0 and S0 modes in this direction. Note that the specific angles chosen are for illustration only—the radiation patterns vary substantially with angle and configuration. Thus, for example, the presence of the SH0 mode only in Figure 16d is only true for the angle of 0° illustrated for configuration 4, and not for any other angle. Actually, for angles greater than 30°, the S0 mode is more dominant. This underlines the fact that great care must be taken in choosing the correct angles if this is required to generate the maximum energy in a specific direction while minimizing the presence of other modes. The waveforms also illustrate that SH0 and S0 modes can be generated at similar magnitudes, but only at different angles and only if the correct configuration is adopted (see, for example, Figure 16a,d).

The amplitude of the A0 mode is lower in all the cases illustrated in Figure 16, but is at a reasonable level for configuration 2 at 90°. This corresponds to the directivity plot of Figure 15, where there is a strong lobe of the A0 mode in that direction. Note also that the A0 mode tends to have greater out-of-plane than in-plane motion at the surface of a plate, and the mechanisms of generation described here tend to favour forces that are in-plane. Hence, the A0 mode is expected, in general, to be less-efficiently generated by magnetostrictive patches of the type considered in this paper.

## 4. Discussion

It is evident that some interesting interactions are taking place within the magnetostrictive patch when attached to a plate. Configuration 1 created orthogonal *B_s_* and *B_d_* fields in the X-Y plane. With no Lorentz force expected, the motion of the patch would be due to magnetostriction effects only. The result was an initial asymmetric wave motion, visualised over the patch, which then led to a complicated directivity pattern for both S0 and SH0 modes. In contrast to this, the A0 mode directivity contained prominent lobes along the 35°–215° and 320°–140° axes. Configuration 1 shows that using magnetostriction forces only to generate guided waves is a complex phenomenon. A full understanding of the underlying complex generation mechanisms at work is outside the scope of this experimental study, and would require modelling of both the interaction between static and dynamic fields and the subsequent guide wave generation from the resultant induced stresses on a particular sample.

Configuration 2 also led to a complicated S0 and SH0 behaviour. However, this configuration could be used to optimise A0 mode generation, while noting that observed amplitudes were smaller than those of the SH0 and S0 modes, whose arrivals can still be observed on waveforms (see Figure 16b). The A0 was generated with bipolar directivity lobes centred along the 90°–270° axis, which was also the direction of both the static and dynamic magnetic fields. Note that rotation of the coil/magnet arrangement relative to the stationary patch would allow the A0 mode to swept at different directions across a plate, making it a convenient method for scanning and defect detection purposes.

In Configuration 3, both Lorentz and magnetostrictive forces were generated in the XY plane. The radiation patterns of A0, S0 and SH0 are all fairly uniform across all angles, as would be expected from the cylindrical geometry of the excitation fields.

Configuration 4 demonstrated that magnetostrictive effects led to a dominant SH0 mode in the expected X direction (0°) direction, due to the orientation of the static field *B_s_*. Thus, this configuration could be used if this mode only is of interest in a particular direction. The A0 mode was generated with a fairly low amplitude and this is due the fact that the A0 mode requires some component of out-of-plane motion (i.e., in the Z direction) which was not favoured in this configuration. The Lorentz forces in theory would be minimal in the 0° and 180° and start to gradually increase toward the 90° and 270°. This can be observed through the polar radiation of the A0 mode, where it is minimal in 0° and 180°.

It should be noted that the measurements were taken using the laser system around 360° without interpolation between measurement points. Note also that the directivity patterns measured for different wave modes in Figure 14 and Figure 15 show some planes of symmetry, as might be expected, indicating that the directivity patterns were measured in a robust manner.

## 5. Conclusions

The use of a vibrometer detector has been shown to allow the characteristics of guided waves generated by removable magnetostrictive patches to be measured for the first time, and some interesting phenomena were observed. It has been demonstrated that these devices can be used to generate S0-, A0- and SH0-guided wave modes with the provision of control capability in terms of directivity and relative amplitude of the three GW modes investigated. The relative amplitude and direction of each of these modes can be altered by changing the dynamic and static magnetic field distributions within the patch, and this has been achieved by using different combinations of dynamic coil shapes and static magnetic field directions. It is evident from the data that there is a complicated interaction between the relative directions of static and magnetic fields and the patch, and that this arises because both Lorentz and magnetostriction forces could be generated, depending on the exact geometry chosen. This interaction is obviously complex, and hence careful selection of the direction of the dynamic and static magnetic fields should be adopted in order to favour specific modes compared to others. A fuller understanding and modelling of the generation mechanisms, and their effect on the resultant guided wave mode properties, is the subject of current research. However, such transducers are likely to find applications in many areas where ultrasonic guided waves are useful, especially in non-destructive evaluation measurements. The ability to adjust the excitation conditions so as to change both the guided wave directivity and the dominant mode for a given patch, simply by changing the magnetic field conditions, will provide great flexibility in such measurements.

The work reported in this paper shows that by adopting such an approach, a particular guided wave mode can be generated in a particular direction with the optimum signal level. This is of advantage in certain measurements where a particular mode is required (e.g., the SH0 mode at 0° for configuration 4). Conversely, configuration 3 provides a fairly uniform radiation pattern for both the S0 and SH0 modes. Once the patch has been attached to the surface, the amplitude of each of the three modes can be adjusted simply by altering the coil and/or magnet geometry, giving great flexibility—a different patch need not be used on the surface. It was shown that the A0 mode is the least favoured by the technique, due to the fact that it requires a large out-of-plane component to be generated efficiently.

## Figures and Tables

**Table 1:** Mechanical and magnetic characteristics of the magnetostrictive patch material (VACOFLUX iron-cobalt alloy).

Young’s Modulus	200 GPa
Poisson Ratio	0.29
Density	8.12 g/cm^3^
Electrical Resistivity	0.42 µΩm
Saturation Magnetostriction	70 ppm
Saturation Magnetisation	2.35 T

**Table 2:** Summary of the configurations used to study different magnetic field combinations.

Configuration	Static Magnetic Field *B*_s_	Dynamic Magnetic Field *B_d_*	Coil Shape
Configuration 1	*X*-axis	*Y*-axis	Racetrack
Configuration 2	*X*-axis	*X*-axis	Racetrack
Configuration 3	*Z*-axis	Radial (X, Y plane)	Pancake
Configuration 4	*X*-axis	Radial (X, Y plane)	Pancake

**Table 3:** Expected velocity of the three guided-wave modes at a frequency of 200 kHz.

Guided Wave Mode	Velocity (m/s)
S0	5146
SH0	3046
A0	1950

Note that rotating the patch by 90° had little effect on the directivity patterns (keeping the coordinate system the same in each case). This seemed to indicate that material anisotropy and/or any preferred magnetostriction direction within the patch was not affecting the results.
